# Social capital and Internet use in an age-comparative perspective with a focus on later life

**DOI:** 10.1371/journal.pone.0192119

**Published:** 2018-02-26

**Authors:** Barbara Barbosa Neves, Jaime R. S. Fonseca, Fausto Amaro, Adriano Pasqualotti

**Affiliations:** 1 School of Social and Political Sciences, The University of Melbourne, Melbourne, Australia; 2 Centro de Administração e Políticas Públicas, Instituto Superior de Ciências Sociais e Políticas, Universidade de Lisboa, Lisbon, Portugal; 3 Gerontology, Passo Fundo University, Passo Fundo, Brazil; Institut Català de Paleoecologia Humana i Evolució Social (IPHES), SPAIN

## Abstract

Older adults (aged 65+) are still less likely to adopt the Internet when compared to other age groups, although their usage is increasing. To explore the societal effects of Internet usage, scholars have been using social capital as an analytical tool. Social capital pertains to the resources that are potentially available in one’s social ties. As the Internet becomes a prominent source of information, communication, and participation in industrialized countries, it is critical to study how it affects social resources from an age-comparative perspective. Research has found a positive association between Internet use and social capital, though limited attention has been paid to older adults. Studies have also found a positive association between social capital and wellbeing, health, sociability, and social support amongst older adults. However, little is known about how Internet usage or lack thereof relates to their social capital. To address this gap, we used a mixed-methods approach to examine the relationship between Internet usage and social capital and whether and how it differs by age. For this, we surveyed a representative sample of 417 adults (18+) living in Lisbon, Portugal, of which 118 are older adults. Social capital was measured through bonding, bridging, and specific resources, and analyzed with Latent Class Modeling and logistic regressions. Internet usage was measured through frequency and type of use. Fourteen follow-up semi-structured interviews helped contextualize the survey data. Our findings show that social capital decreased with age but varied for each type of Internet user. Older adults were less likely to have a high level of social capital; yet within this age group, frequent Internet users had higher levels than other users and non-users. On the one hand, the Internet seems to help maintain, accrue, and even mobilize social capital. On the other hand, it also seems to reinforce social inequality and accumulated advantage (known as the Matthew effect).

## Introduction

As identified by several organizations, the social challenges of an aging and aged population directly affect older adults–not just their communities [1]. For instance, social exclusion, social isolation, and loneliness are becoming emerging issues in later life–negatively affecting the health, wellbeing, and social participation of older adults [2,3]. Research has shown that the Internet can help address issues of social participation, connectedness, and well-being by providing: 1) communication opportunities with a variety of social ties (strong, weak, new, latent, etc.) regardless of geographic location, which can minimize mobility limitations and alleviate social isolation and loneliness, and 2) information and services (e.g., health-related information, online shopping, banking, entertainment, etc.) that can facilitate activities of daily living, social participation, and active ageing [4–12].

Internet use among older adults, however, still lags behind other age groups despite increasing in the last years. In the 28 countries of the European Union, 26 per cent of older adults (aged 65–74) use the Internet frequently compared to 88 per cent of people aged 16–24 [13]. In the US, 59 per cent of older adults use the Internet, but only those who are younger, higher-income, and more highly educated use it at rates that approach the general population [14]. As such, Internet usage relates to social inequalities not only in terms of age but also in terms of education and social status. Older adults without access or the skills to use the Internet are missing out on resources for social connectedness, support, and participation. These resources can be captured by the social capital concept, which encompasses the resources embedded in our relationships, representing a health and psycho-socio-economic asset for older adults [5,15–17]. Previous research has identified a positive association between social capital and Internet use among adults and young adults [18]; however, it is unknown whether and how this association plays out among older adults or differs by age.

In a society where the Internet is becoming a social resource (for example, in 2014, 78 per cent of EU citizens, aged between 16 and 74, used the Internet for networking and information seeking [19]), we draw on a representative sample of adults to examine social capital and Internet use in old age and across different age groups. Combining survey data and qualitative interviews, our research contributes to an understanding of the critical relationship between social capital and the Internet and sheds further light on age dynamics.

## Social capital, Internet, and older adults

### Defining social capital

Social capital is a widely-used sociological construct to capture the value of our social relationships [16]. However, the recent uptake of social capital across disciplines and its broad nature has led to conceptual, theoretical, and operational ambiguities. These include different definitions of social capital (e.g., some focus on social ties and resources, while others on civic engagement and trust) and levels of analysis (individual, community, or nation-state). Due to these ambiguities, social capital has received much criticism, leveled, in particular, at its wide application as an umbrella term [20,21]. But even critical authors recognize the scientific value of social capital to enclose resources embedded in social relationships, and to bridge structure and agency in the study of social networks and social inequality [16,21–24]. To overcome ambiguities, researchers must advance clear definitions, consistent operationalizations, and acknowledge different sources and effects of social capital [21,22].

Social capital is not a new sociological concept, having existed as an idea in the work of Émile Durkheim and Max Weber, among others [21,25]. Although Lyda Hanifan (1916) is usually credited as the first known author to use the term social capital in a modern sense, recent historical research shows that John Dewey (1900) used the concept earlier [25]. Pierre Bourdieu [26] and James Coleman [27] developed the contemporary definition of social capital, although in different directions. Bourdieu [26,28] saw social capital as a result of resources embedded in social networks and investments in those social networks; Coleman [27,29] defined it by function, i.e., by its uses and effects. For Coleman, social capital “is not a single entity but a variety of different entities, with two elements in common: they all consist of some aspect of social structures, and they facilitate certain actions of actors–whether persons or corporate actors–within the structure” (p98 in [29]). Coleman’s wider definition adds to the ambiguity outlined earlier. More recently, Robert Putnam [30,31] popularized social capital in the study of civic life and communities (macro level). For Putnam [31], social capital includes social connections, networks of civic engagement, norms of reciprocity, and trustworthiness.

The common elements of the various definitions of social capital are *relationships* and *resources*–be it at the micro, meso, or macro level [32–34]. Our relationships matter and give us access to a range of resources that can be used for personal and collective gain [22]. But social capital is not always a public good, as suggested by Coleman [29] and embraced by more contemporary authors [31]: using a well-known example, the Ku Klux Klan displays high levels of social capital amongst its members, albeit with negative societal outcomes, such as oppression, discrimination, exclusion, in-group mentality, etc. [32]. At the micro level, social capital (as with other forms of capital) is mainly considered a positive resource for the individual or group being analyzed [22]–even if reproducing broader social inequalities and excluding other individuals or groups [28]. In fact, as shown by Bourdieu [26,28], social capital is relational: it interplays with other forms of capital (cultural, economic, and symbolic), dynamically connecting agency and structure.

Since we are interested in this relational perspective and in an individual-analytical level (micro and meso), we draw on Bourdieu’s definition of social capital: “it is the sum of actual or potential resources related to the possession of a durable network of more or less institutionalized relationships of acquaintance and recognition” (p2 in [26]). In other words, social capital captures the resources that are potentially available in our social networks (e.g., family members, friends, neighbors, acquaintances, etc.). These resources include social, psychological, economic, political, cultural, and symbolic assets, which can be employed for instrumental (e.g., help finding a job) or expressive (e.g., emotional support) actions [23]. Instrumental actions are mostly taken to gain resources, whereas expressive actions to maintain resources [23]. As such, action and social structure are embedded in social capital theory: motivated actions lead to specific interactions in a social network, but the mobilization of resources is controlled by the availability of those resources and by the diversity of the social structures wherein individuals act [23].

Our definition of social capital does not include social norms, trust, or civic engagement, as considered by Coleman and Putnam. This is because a body of research has shown non-existent or marginal empirical associations between social capital and norms, civic engagement, and social trust, suggesting that although these might be related, they are independent concepts [16]. Additionally, much of the criticism on social capital rests on the extension of the concept by Putnam [31] to include “civicness” and be “a feature of communities and even nations” (p18 in [21]), rendering the concept tautological and heuristically weak [22,35,36].

### Social capital and the Internet among older adults

Social capital matters for older adults, because it has positive effects on health, wellbeing, social support, sociability, and social standing [15–17]. Older adults with higher levels of social capital are healthier and engage in healthier behaviors [17], and display better physical and social wellbeing even when single or having low-income [15]. Social capital, namely its institutional or formal dimension, also affects the health of older adults (60+) more intensely than younger adults [35]. And as with other types of capital–economic, cultural, and symbolic–those with higher levels of social capital tend to have greater social and economic opportunities [16,28]. Social capital is not only a useful tool to study social connectedness and wellbeing in old age, but has also been invaluable in studying the social impact of the Internet [33,34]. While various studies examine the relationship between the Internet and social capital, particularly among young adults [37–39], relatively little attention has been paid to older adults or different stages of adulthood.

The existing literature on the subject, scant as it is, suggests that Internet use among older adults relates to higher levels of social capital [40,41]. Considering general Internet use among Australian older adults (n = 222, 55+), Sum *et al*. [41] conclude that long-term Internet users had more probability of high family connections (known as bonding social capital) and ranked higher in acceptance of diversity (known as bridging social capital). These authors also demonstrate that changes in social capital remained constant after a certain frequency of Internet use, with the exception of using the Internet for communication [41]. One may argue that online entertainment and commerce are more individualist online activities, limited in their long-term impact on social capital. Similarly, Russell and colleagues’ study [40] of an online sample of Australian older adults (n = 154, 55+) determined that online communication (emailing) increased satisfaction with family communication, allowed older adults to grow their close networks, and led to in-person interactions. Other online activities, such as general browsing, online shopping, etc., did not elicit the same results as emailing. Despite providing significant data on social capital and the Internet in late adulthood, both studies are based on convenience samples of frequent Internet users with a sizable proportion of adults (55–64 years of age) limiting, therefore, the scope and representativeness of their results.

Exploring a specific online communication medium and social capital, Erickson [42] interviewed seven older users of Facebook (a popular social networking site), and ascertained that it was mostly used to know what was going on with loved ones and not for substantial interaction. This awareness would, however, induce face-to-face or telephone communication. Facebook might be facilitating maintenance and mobilization of social capital through that awareness. Although exploratory, this study raises questions about different roles of online media (for instance, email vs. social networking sites) on social capital. Likewise, Zhang and Kaufman [43] concluded that collaborative online gaming is positively associated with social capital amongst older adults (55+) when players enjoy their relationships and gameplay context. Though growing in popularity, this type of online activity is still marginal and highly selective in later life, as around 2% of players are aged 60+ [43].

Taking a different direction, Choi and DiNitto [5] explored whether psychological and social capital could predict Internet use and types of usage among American older adults. They show that social capital was positively associated with Internet use, but older adults who used the Internet solely for emailing were older and the socially disadvantaged group of Internet users. Internet users were, nevertheless, more advantaged in terms of social support and participation, and in better health than non-users. Some health problems can limit or enhance Internet use among older adults: the authors found that dementia was the only health status variable negatively associated with Internet use (due to its cognitive and functional impact), whereas having more chronic medical conditions was positively associated with using the Internet for health-related activities (e.g., to get information about health conditions, to contact a medical provider, to deal with health insurance, etc.). Older adults who reported depressive or anxiety symptoms were also less likely to use the Internet. This study provides representative data and valuable insights into the social capital-Internet dyad, but the indicators of social capital were limited to dichotomous variables on having one child/stepchild, having one living sibling (assuming these were strong ties), and social participation.

In brief, empirical evidence on the relationship between Internet use and social capital in late adulthood is scant. The few existing studies are mostly quantitative, based on convenience samples, and do not use a mixed-methods or a qualitative approach to better contextualize the lived experiences of older adults regarding social capital and Internet use/non-use. We also lack an age-comparative viewpoint, drawing on a life course perspective, to help us fully understand the role of the Internet in old age. Although this article focuses on later life–having older adults as the baseline group–to ensure that age-based comparison we asked:

RQ: How does Internet usage and age relate to social capital?

To answer this research question, we surveyed a representative sample of 417 adults (18+) living in Lisbon, Portugal, of which 118 were older adults (S1 File). This survey research was complemented by 14 semi-structured interviews, used to explore social circumstances and meanings around social capital and the Internet.

## Methods

### Sample and data collection

We surveyed a representative stratified random sample of 417 inhabitants of Lisbon, Portugal. Lisbon is the capital of Portugal and its most populated city: according to the 2011 census, it had 547,733 inhabitants [44]. Portugal has a population of 10.5 million, 48 per cent male and 52 per cent female; 14.7 per cent of people aged 0–14, 65.7 per cent of people aged 15–64, and 19.6 per cent of older adults [45]. In line with other European countries, Portugal has an ageing population–in 2014, the national ageing index (the number of people aged 60+ per hundred people under age 15) was 141 per cent and the Lisbon index was 189 per cent [46]. It also has one of the lowest birth rates in Europe: 7.9 at the national level, 10.6 in Lisbon [46]. Culturally and ethnically, Portugal is fairly homogeneous and the majority of the population is of European origin [47]. Portugal shares demographic and general social capital patterns with other European countries, particularly Southern countries [48,49].

The survey sample was drawn out of the 53 administrative local councils that comprised the city of Lisbon in 2010. We then defined four strata according to the number of inhabitants. Within each stratum, we randomly selected the councils to define the sampling points. We used quota sampling by gender and age, in the final phase of the sample design, to proportionally map the demographic structure of Lisbon according to the 2001 census data [50]. As such, we had four age groups: 18–34; 35–44; 45–64; 65+.

The questionnaires were administered face-to-face in the households of the interviewees, during March and September of 2010, and only one adult person per household was interviewed. To select the households, we used the random route technique, which randomizes dwellings, and a table of random numbers to select the floor and/or unit if applicable. In each selected household, we recruited one participant according to our age and gender quotas. All participants were recruited through door knocking and were assured confidentiality and anonymity in the consent forms and by the interviewers. The University and research center did not require formal IRB approval, as common in social sciences research conducted in Portugal and other European countries (S2 File). However, the *International Sociological Association’s* Code of Ethics was strictly followed. Our selection criteria excluded people with health-related issues that could prevent them from giving informed consent, such as cognitive impairments (self-identified). Nineteen trained interviewers administered the questionnaires. The survey yielded information about social networks, sociability, social capital, and Internet use.

Of the survey respondents, 54 per cent were female and 46 per cent were male. Mean age was 50.1 (*S*.*D*. = 19.5; Median = 49), and the age of the participants ranged from 18 to 93 (see Table 1 for all age groups). The sample was composed of 102 respondents aged 18–34, 61 respondents aged 34–44, 136 respondents aged 45–64, and 118 respondents aged 65+. Of our baseline group, the sub-sample of older adults (n = 118), 57 per cent were female and 43 per cent male, and most were retired (87%), had less than secondary education (80%), were married/de facto (61%) or widowed (31%), and lived with their partners (49%), alone (25%) or in other arrangements (21%). Of the youngest group, 58 per cent were female and 42 per cent male, and most were employed (55%), had secondary education (41%) or less than secondary education (35%), were single (81%), and lived with their partners (59%). Considering the group aged 34–44, 54 per cent were female and 46 per cent male, most were employed (90%), had less than secondary education (51%), were married/de facto (57%), and lived with their partners and children (56%). Finally, regarding the 45–64 age group, 49 per cent were female and 51 per cent male, and most were employed (81%), had less than secondary education (65%), were married/de facto (74%), and lived with their partners and children (55%).

**Table 1:** Sample characteristics (%, aged 18+).

**Gender**	Female	54.1
	Male	45.7
**Age**	18–34	24.4
	35–44	14.6
	45–64	32.5
	65+	28.2
**Marital Status**	Single	28.5
	Married/De facto	54.1
	Divorced/Separated	6.2
	Widowed	11
**Household Type**	One-Person Household	17
	Couple without children	24.2
	Nuclear family	42.3
	Other	16.3
**Occupation**	Employed	56
	Unemployed	4.1
	Student	9.6
	Retired	26.1
	Housewife	2.9
**Education**	No education	1.7
	Less than secondary education	57.9
	Secondary education	23.4
	University degree	13.6
	Master/PhD	2.9

Note: Total percentages may not add up to 100, owing to rounding errors or missing values.

To deepen and contextualize the quantitative findings we also conducted 14 follow-up semi-structured interviews, in a sequential mixed methods design. At the end of the questionnaire, we asked whether respondents would be willing to participate in a follow-up interview; if yes, we requested their contact information. The number of qualitative interviews was determined by the available contacts for a follow-up, and by the representativeness/saturation trade-off–i.e., a balance between the requirements for representativeness of quantitative sources and saturation of qualitative information [51]. Eight interviewees were female and six were male. Age ranged from 26 to 75 years (Mean = 50.7, *S*.*D*. = 19.8). Five had secondary education, seven had undergraduate education, and two had a postgraduate degree. Of the 14 interviewees, six were older adults (Mean = 75.2, *S*.*D*. = 7.7). The interviews were conducted from January to March 2011, each lasting an average of 40 minutes. All interviewees provided additional written informed consent and chose the pseudonyms used throughout this article.

### Measures

#### Social capital

Matching our definition of social capital, we considered the two main dimensions of social capital: bonding and bridging, as coined by Ross Gittell and Avis Vidal [52] and popularized by Putnam [31]. While we are critical of Putnam’s stance on social capital, we found it fruitful to draw on these dimensions, particularly as they have been improved upon by several authors as a response to previous criticism, and continue to be central in social capital studies [53]. Bonding relates to the resources that are potentially available in our strong ties (e.g., family members, close friends, confidants) and that can be mobilized when needed. Because strong ties are usually similar in terms of social backgrounds, bonding is mostly an instrument for expressive actions [23]. Bonding social capital is the main provider of social support, ranging from emotional to financial aid. Bridging relates to the resources that are potentially available in our weak ties (e.g., acquaintances). Because weak ties are usually more heterogeneous than strong ties, bridging is an optimal tool for instrumental actions [23]. For example, weak ties have access to different and more various resources, such as information on job leads [54,55] and broader social perspectives [56]. Strong ties can also facilitate bridging (e.g., a friend of a close friend), but bridging is mainly possible through weak ties [57].

So to measure social capital we need to capture both the quantity and quality of social ties, strength of relationships indicating readiness to help, and availability and type of resources [58]. Thus, we measured bonding and bridging by combining a group of indicators and looked at a set of resources using the resources generator (see Table 2). Among that group of indicators, previously validated in social capital research [48,59–62], are items from the *Internet Social Capital Scales* (ISCS) that consider offline and online bonding and bridging dimensions [56]. The bonding items tap into emotional and social support, whereas the bridging items tap into broader worldviews and opportunities for new resources [56]. For this article, we only considered the offline bonding and bridging sub-scales (see Table 2). The difference between offline and online sub-scales is that the online items are specific to “online” interactions: for instance instead of statements such as “interacting with people makes me…”, the online sub-scales state “interacting with people online makes me…”. Although we measured these online sub-scales–which assess online interaction and its value–it confused our pre-test respondents (n = 30) since most of their offline ties are also online ties: the friends and relatives that they communicate with online are also the friends and relatives that they communicate in person and through other media. To avoid data redundancy, for the online dimension of social capital we considered people that the respondents exclusively knew online (and not in person). Our aim was to create a broad social capital variable with the online and the offline dimensions, but our sample only had 28 per cent of respondents that knew “only online” people (i.e., people that they never met in person). With such significant reduction of cases (109 instead of 417), we decided to consider only the offline dimensions. Despite this setback, we believe our social capital variable includes enough indicators to provide a comprehensive construct. The qualitative data also helps to enhance this comprehensiveness.

**Table 2:** Dimensions and indicators of social capital.

**BONDING**
1. Three items of the *Bonding Sub-scale*, 5-point Likert scale from strongly disagree to strongly agree [56]: • I do not know people well enough to get them to do anything important (reversed) **(Bonding1)** • When I feel lonely, there are several people I can talk to **(Bonding2)** • If I need any help to solve my problems, I know several people available to help me **(Bonding3)**
2. Number of close relatives
3. Frequency of contact (Face-to-face/Telephone/Mobile phone/Internet; 1 = daily; 2 = at least once a week; 3 = at least once a month; 4 = rarely/never)
4. Number of close friends
5. Frequency of contact (Face-to-face/Telephone/Mobile phone/Internet; 1 = daily; 2 = at least once a week; 3 = at least once a month; 4 = rarely/never)
**BRIDGING**
1. Three items of the *Bridging Sub-scale*, 5-point Likert scale from strongly disagree to strongly agree [56]: • Interacting with people makes me interested in different ideas **(Bridging1)** • Interacting with people makes me feel connected to the bigger picture **(Bridging2)** • Interacting with people makes me want to try new things **(Bridging3)**
2. *Social diversity*, 5-point Likert scale from strongly disagree to strongly agree [48,59]: • I’m interested in people with different life styles
3. *Informal networks*, by frequency: 1 = daily; 2 = at least once a week; 3 = at least once a month; 4 = rarely/never [60]: • In the last month, I went out socially with my friends (friends in general, not considering close friends)
**RESOURCES**
1. Do you know anyone who…? (Items from the *Resources Generator*, adapted from Van der Gaag and Snijders [61]; coded as 1 = family, 2 = friends, 3 = neighbours, 4 = co-workers, 5 = acquaintances, 6 = none, and 7 = other): • Can help with small jobs around the house • Can provide a place to stay if you have to leave your house temporarily • Can give advice on matter of laws and regulations • Can help you if you need to find a job • Can help you if you need to use a computer/go online • Can help you if you need anything from the municipal parish/local government

To maximize our construct coverage (as we included different instruments usually administered on its own) and to avoid a lengthy and repetitive questionnaire, we did not use the 33 items of the resources generator or the 40 items of the ISCS. We selected items based on analytical and cultural relevance, factor loading estimates, and average inter-item correlations of original and subsequent applications of the scales (see Table 2 for selected items and coding). All the items and additional indicators used were then combined to define the dependent variable social capital, through Latent Class Modeling (LCM) that takes into account reliability and internal consistency of the used items.

#### Internet

Internet usage, our main independent variable, was measured through frequency of usage and grouped into non-users, light users, moderate users, and heavy users. Light users correspond to the respondents that use the Internet at least once a month or rarely; moderate users correspond to the respondents that use the Internet 1 to 4 times a week; and heavy users correspond to the respondents that report using the Internet daily. Besides general Internet usage, we added three secondary independent variables related to type of use: email, instant messaging (IM), and social networking sites (SNSs). These were categorized as using or not using these services (dichotomous), and allowed us to examine if a more social-driven or specific type of Internet usage could differently impact social capital.

#### Socio-demographic variables

Age was our main demographic variable, but we also controlled for other socioeconomic variables used in social capital studies, including gender, marital status, education, occupation, religion, and household composition.

### Data analysis

The quantitative data were analyzed using descriptive statistical analysis, correlations, and binominal logistics regressions on IBM SPSS Statistics 18 (*Forward*:*LR* method), and applying Latent Class Modeling (LCM) on LatentGold 3. The variable social capital was estimated with LCM–a cluster analysis technique that creates latent classes from multivariate data by identifying classes that explain the associations between a set of observed variables and by distributing the observations among those classes [63]. LCM is the most appropriate technique for the type of data collected, because: 1) it does not rely on conventional modeling assumptions (such as linear relationship, normal distribution, homogeneity, etc.); 2) works with categorical, continuous, and mixed variables; 3) offers a robust clustering while combining latent classes and external variables (e.g., socio-demographics) with no need for discriminant analysis; and 4) is unaltered by linear transformations of variables [64,65]. Consequently, social capital is the latent variable we wanted to estimate through the multiple *observable indicators* (i.e., the set of variables used to measure bonding, bridging, and resources). LCM defines classes with these indicators, which means that respondents with similar attributes and behaviors are grouped into the same class. So different classes display the heterogeneity of attributes and behaviors of respondents, helping to categorize them in different levels of social capital. After estimating the social capital variable, we ran binomial logistic regressions to measure the effect of the Internet (frequency of usage and different types of usage) on the probability of having a low or high level of social capital. Because of the strong correlation between age and Internet use, we added an interaction term to our model (age*Internet) and controlled for the aforementioned sociodemographics.

The semi-structured interviews were audio-recorded and transcribed (verbatim) before being coded. The interviews were conducted in Portuguese and its reporting in English may affect meanings and, therefore, the qualitative analysis. To minimize this risk, the interviews were analyzed in the original language and the translation of quotations was carefully done to consider their readability in English while respecting the Portuguese utterance. The qualitative data were analyzed with profiling and thematic analysis: qualitative profiling allowed us to craft individual profiles for each interviewee considering their perspectives and contexts, while the thematic analysis allowed us to examined categories, patterns, and connections, trying to find a balance between within-case and cross-case analysis [66,67].

## Results

### Internet use and non-use

Our sample had 37 per cent of Internet non-users and 63 per cent of Internet users (n = 417). These results are similar to national and European statistics: in the same period, 55 per cent of Portuguese people used the Internet [68] and 69 per cent of European Union citizens used the Internet [69]. Considering different levels of Internet use, we divided our respondents into four groups:

1. Non-users (37%);
2. Light users (3.1%)–used the Internet at least once a month or rarely;
3. Moderate users (12.9%)–used the Internet 3 or 4 times a week or 1 or 2 times a week;
4. Heavy users (47%)–used the Internet daily.

Non-users were mainly older adults (62%), whereas Internet users were concentrated around the younger groups: 39 per cent of users and 0.5 per cent of non-users were between 18–34 years old (see Table 3). Our non-users were also mostly retired or inactive and less educated. In fact, Internet usage is negatively correlated with age (*rs*(417) = -0.707, *p* ≤ 0.001), but positively correlated with education (*rs*(416) = 0.483, *p* ≤ 0.001). Internet usage and gender were not significantly correlated (*rs*(417) = 0.049, *p* = 0.323).

**Table 3:** Types and Internet use by age groups.

Types & Internet Use	Age Groups (% within age group)			
	18–34	35–44	45–64	65+
Non-user	1	9.8	38.2	85
Light-user	2	1.6	4.4	3.4
Moderate-user	8.8	19.7	16.9	8.5
Heavy-user	88.2	68.9	40.4	7.6
Emails	68.3	80.4	78.6	72.7
Uses IM	57.4	39.3	25	27.3
Uses SNSs	42.6	19.6	22.6	13.6

The main reasons for Internet use by our overall sample were: to search (44%), to study (26%), and to talk to family and friends (13%). However, the main online activities (multiple-response question) were to send/receive emails (29%); to browse websites (28%); to use instant messaging services or similar services (16%); and to use social networking sites (11%)–these were similar across the age groups. Our older Internet users reported using the Internet to search (81%) and for leisure (19%), but their main online activities were emailing (73%) and browsing websites (73%). All age groups (35+) emailed more than young adults (see Table 3). Of the 93 per cent of global email users, 53 per cent mostly sent emails to friends, 27 per cent to co-workers/colleagues, and 7 per cent to family members. Older adults reported sending more emails to relatives than other age groups and more emails to friends than participants in the 35-64-age range. We found no significant correlations between reasons to use the Internet, online activities, emailing and our socio-demographics. Only 44 per cent of our Internet users reported meeting people online, which accounted for 5 per cent of older adults. The 18–24 age group reported meeting more people online than any other group (61%). A point-biserial correlation showed that meeting people online is negatively correlated with age (*r*_pb_ (263) = -0.296, *p* ≤ 0.001).

Of the general 73 per cent of respondents that used instant messaging services (IM), mostly young adults (57%, see Table 3), such as *Microsoft Messenger*, most used it to talk to friends (63%). The same reason was advanced by the 27 per cent of older adults that used IM (and other age groups). However, as with email, a higher percentage of older adults used IM to contact relatives than the other age groups. In terms of social networking sites (SNSs), 65 per cent of Internet users had a profile in these sites (77% on Facebook). SNSs were mostly popular within the young age group (42%, see Table 3). Fourteen per cent of older adults reported having a SNS profile, also primarily on Facebook. Overall, SNSs were mainly used to: be in contact with friends (33%), be in contact with colleagues (14%), share ideas and news (13%), and meet new people (11.9%). Age correlates with IM (*rs*(263) = 0.266, *p* ≤ 0.001) and SNSs (*rs*(260) = 0.411, *p* ≤ 0.001).

In terms of device ownership, 93 per cent of our sample reported owning a mobile phone, 62 per cent a laptop, and 30 per cent a personal computer. One hundred per cent of participants aged 18–44 had a mobile phone, followed by 97 per cent of those aged 45–64 and 79 per cent of older adults. Owning a laptop was higher than owning a personal computer for all age groups. Young adults (18–24) reported higher percentages of laptop ownership than any other group (92%); older adults the lowest (20%). Respondents aged 35–44 had the highest ownership of personal computers (46%); older adults the lowest (9%).

Finally, most respondents indicated that the Internet made it easier to be in touch with close relatives and friends (71%) and with other relatives and friends (77%). When we look at disaggregated data, only older adults were not as definitive: 59 per cent and 55 per cent respectively.

### Social capital

#### Descriptive results

More than half of the respondents reported a ‘high’ or ‘positive’ level of bonding on the three items of the *Internet Social Capital Scale* (see Table 4). These levels are similar across age groups, although older adults reported more negative values than others. The number of close family members indicated by the overall sample ranged between 0 and 40, with a mean of 8.07 (*SD* = 5.173; Median = 8; Mode = 10). The number of close friends also ranged between 0 and 40 with a mean of 8.28 (*SD* = 6.572; Median = 6; Mode = 6). Older adults, however, had relatively fewer close relatives (M = 7.84, *SD* = 5.580, Median = 6, Mode = 5) and friends (M = 7.02, *SD* = 6.473, Median = 6, Mode = 6). We found a negative correlation between age and number of close friends (*r*(388) = - 0.149, *p* ≤ 0.001), but no significant correlation between age and number of close relatives.

**Table 4:** Frequencies of bonding and bridging sub-scales (%).

	Bonding1	Bonding2	Bonding3	Bridging1	Bridging2	Bridging3
Strongly disagree	3.6	1.4	0.4	1.4	1	1.4
Disagree	17.3	19.2	7.9	4.8	17.5	7.9
Neither agree, nor disagree	10	20.6	5.8	13.2	38.4	24.8
Agree	61.4	51.1	74.6	69.8	37.1	56
Strongly Agree	7.7	7.7	11.3	10.8	6	9.9

Note: Bonding1 –“I do not know people well enough to get them to do anything important”. (Reversed)

Bonding2 –“When I feel lonely, there are several people I can talk to”.

Bonding3 –“If I need any help to solve my problems, I know several people available to help me”.

Bridging1 –“Interacting with people makes me interested in different ideas”

Bridging2 –“Interacting with people makes me feel connected to the bigger picture”

Bridging3 –“Interacting with people makes me want to try new things”

Most respondents met family face-to-face on a daily basis (62%), and used the mobile phone to contact them daily (59.2%). These values are similar across age groups. Slightly less than half of the respondents met their close friends face-to-face on a daily basis (42.9%), and slightly more than a quarter used the mobile phone daily to contact their close friends (36.7%). A higher percentage of older adults reported meeting their close friends face-to-face on a daily basis (50%), although only 7 per cent used the mobile phone (8% used the telephone) daily to contact close friends. The Internet was less used for frequent communication with relatives: only 15 per cent of the Internet users (and 4% of older adults) interacted daily with their close relatives online and 19 per cent (2% of older adults) did so once a week. But the Internet was frequently used for communication with close friends: 40 per cent reported contacting them online on a daily-basis, 28 per cent once a week, and 4 per cent once a month. Older adults were the only age group indicating use of the Internet more for daily communication with close relatives (4%) than with close friends (1%).

Participants mostly reported a “high” or “positive” level of bridging social capital in two of the three items of the bridging scale (see Table 4). The item *bridging 2* had a higher percentage in the “neither agree, nor disagree” response (38.4%), although the response “agree” followed closely (37.2%). This question might be perceived as too abstract to pin down. Although these percentages are similar across age groups, older adults reported less “strongly agree” than any other group. More than half (63.5%) of the respondents were interested in people with different lifestyles (social diversity); 43 per cent of older adults agreed, although 32% neither agreed nor disagreed. In terms of social participation: slightly less than half reported going out socially with (somewhat close) friends at least once a week (46.3%), while almost a quarter (19.7%) did it on a daily basis. Interestingly, after the youngest age group (34%), older adults (17%) reported going out daily with friends more often than the two middle age groups (15% and 13%, respectively).

Regarding resources, most respondents reported having access to the eight resources. The most available resource was getting *help when sick* (98%), whereas the least available was *help to find a job* (86%). Results on the availability and type of resource were similar across age groups. It seems that resources related to expressive actions are slightly more available than resources related to instrumental actions. Family is the primary source for all resources, with the exception of finding a job. To find a job, people relied more on friends (42.2%) than family (27.3%). Acquaintances got a marginally higher value than friends with regard to being resources with any business at the municipal council/local government (23.5% vs. 22.8%), even though family still dominates as a resource (34.3%). Age and four resources are correlated, namely having a place to stay, advice on matter of laws/regulations, finding a job, and help to use a computer/the Internet (*p* < 0.001). Older people seem to rely more on family for all these resources, while younger people (18–34) seem to rely more on friends for job leads and help with digital technology and on both family and friends for the other two resources. The middle age groups seem also to draw more on family to access resources; apart from the ‘finding a job’ resource, which is mostly supported by friend networks.

#### Estimating social capital

To uncover a general social capital variable we used Latent Class Modeling (LCM) to first estimate each of the three dimensions of social capital through a probabilistic clustering of its indicators. The bonding indicators estimated a two-class bonding social capital (low and high), the bridging indicators estimated a three-class bridging social capital (low, medium, and high), and the list of resources estimated a two-class variable (yes or no). Considering that the selected indicators are categorical, these classes were estimated using the AIC_3_ criterion [64] to determine the optimal number of classes that explain the relationship observed amongst indicators. These classes are based on the attributes of all our respondents (n = 417), allowing us to categorize different levels of bonding, bridging, and resources. The final bonding, bridging, and resources variables were then combined in LCM to find the latent social capital variable, which was estimated with two classes (see Table 5). The first class accounts for 70 per cent of the data, while the second class accounts for 30 per cent of the data.

**Table 5:** Model parameters’ estimates of social capital.

Overall Probability	Class 1	Class 2
Class size	0.6985	0.3015
**BONDING**		
Low	0.4517	**0.5483**
High	**0.964**	0.036
**BRIDGING**		
Low	0.346	**0.654**
Medium	**0.7683**	0.2317
High	**0.9334**	0.0666
**RESOURCES**		
Yes	**0.7576**	0.2424
No	0.3766	**0.6234**

Note: The entries in bold refer to the categories that best characterise each class.

Table 5 shows two kinds of probabilities: first, the ordinary probabilities or proportions of mixture, i.e., the probabilities of belonging to class 1 and class 2, which is 0.70 and 0.30. Second, the conditional probabilities: for instance, 0.4517 and 0.5483 are the probabilities of having “low” in the variable bonding given that the individual belongs to class 1 or class 2; also 0.5483 is higher than 0.4517, so “low” is a characteristic of class 2. This data clustering gives us the profile of the social capital variable, allowing us to differentiate between a lower and a higher level of social capital (see Table 6).

**Table 6:** Profile of social capital.

	High (70%)	Low (30%)
**BONDING**	High	Low
**BRIDGING**	Medium; High	Low
**RESOURCES**	Yes	No

To label these classes, we considered those two levels since we cannot assume that our respondents have no social capital. Class 1 corresponds to high social capital and class 2 to low social capital. Because we cannot quantify the difference between high and low, these were treated as ordinal variables. Having established a social capital variable, in the next section we show the effect of the Internet on the probability of having a low or high level of social capital.

### Social capital, Internet usage, and age

The binary logistic regressions show only three predictors of the relationship between Internet usage and social capital. As seen in Table 7, there is a significant interaction effect between age and Internet usage (*p* = 0.015). When the logistic regression is performed without the interaction term, Internet usage (and not age) is a significant predictor of social capital. This means that the effect of the independent variable age on the dependent variable social capital is different at distinctive levels of the independent variable Internet usage or vice versa. So, we must consider the effect of age on the odds of having a high social capital for a fixed level of Internet usage. Marital status (*p* = 0.015) and household (*p* = 0.001) also had a statistically significant effect on the Logit of the probability of social capital.

**Table 7:** Logit coefficients of the logistic regression model of social capital.

	*B*	S.E.	Wald	df	Sig.	Exp(B)
Age*Internet			10.477	3	0.015	
Age by Internet(1)	-0.017	0.007	5.958	1	0.015	0.983
Age by Internet(2)	-0.046	0.016	7.911	1	0.005	0.955
Age by Internet(3)	-0.010	0.010	0.932	1	0.334	0.990
Marital status			10.552	3	0.015	
Marital status(1)	3.552	1.168	9.252	1	0.002	34.866
Marital status(2)	0.173	0.812	0.045	1	0.831	1.189
Marital status(3)	1.197	0.824	2.111	1	0.146	3.310
Household			12.881	3	0.005	
Household(1)	-2.129	0.648	10.799	1	0.001	0.119
Household(2)	-0.614	0.724	0.720	1	0.396	0.541
Household(3)	-0.012	0.721	0.000	1	0.987	0.988
Constant	2.115	0.709	8.894	1	0.003	8.293

Notes:

I. The model is significant (G^2^ (9) = 80.830; *p* ≤ 0.001) and fits the data well, according to the Hosmer and Lemeshow test (x^2^_HL_ (7) = 5.850, *p* = 0.577). The pseudo R-squares are: R^2^_N_ = 34%, R^2^_CS_ = 21%. This fitted model classified correctly 85 per cent of the cases: sensitivity was 97.9 per cent and specificity was 29.5 per cent, which shows that the classification of this fitted model was proportionally higher than a classification obtained by chance. Despite the relatively low specificity, the ROC Curve analysis presents an excellent discriminant capacity (ROC *c* = 0.812; p ≤ 0.001).

II. Variables

- Internet(1) = Non-user; Internet(2) = Light user; Internet(3) = Moderate user; Baseline = Heavy user.

- Marital status(1) = Single; Marital status(2) = Married/De facto; Marital status(3) = Divorced/Separated; Baseline = Widowed.

- Household(1) = One-person households; Household(2) = Couples without children; Household(3) = Couples with children; Baseline = Other household type.

The interaction term age*Internet has two significant response categories: age by Internet(1), which corresponds to the non-user category and the age by Internet(2), which corresponds to the light user category. These two categories are statistically significant (*p* = 0.015 and *p* = 0.005) comparing to the reference category heavy Internet users. Hence, the effect of age on social capital depends on different values of Internet usage:

- Per year, the odds of having a high social capital decreased for Internet non-users (comparing to heavy users) multiplicatively by a factor equal to *e*^(-0.017)^ = 0.983, or by 1.7 per cent.
- Per year, the odds of having a high social capital decreased for Internet light users (comparing to heavy users) multiplicatively by a factor equal to *e*^(-0.046)^ = 0.955, or by 4.5 per cent.

**Fig 1:**
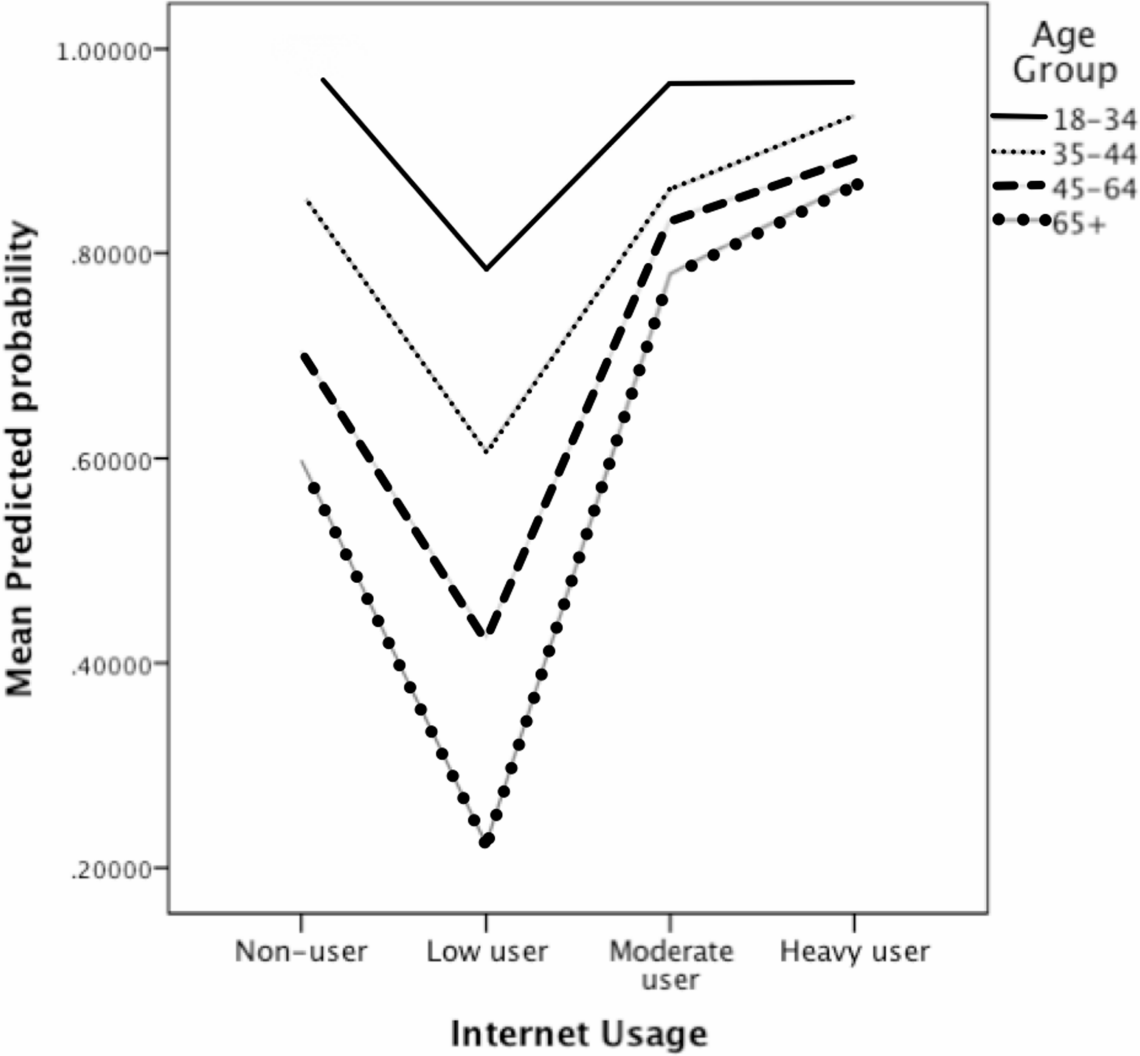
Mean predicted probabilities of social capital by age group and Internet usage.

Fig 1 shows that the older age group had significantly lower odds of high social capital for any type of Internet usage. Interestingly, regardless of age, light users had less probability of having high social capital than non-users and other user-types. However, young light users were more likely to have a high level of social capital than old light users. Heavy Internet users had more probability of having high social capital for all age groups.

Additionally, single people had higher odds of a high level of social capital comparing to widowed people; people living alone had higher odds of high social capital comparing to people in other household types.

The qualitative interviews helped to contextualize these results: of our 14 interviewees, seven were heavy Internet users, four were moderate Internet users, and three were non-users (see Table 8). The non-users were older adults (age ranging from 74 to 85), although we also had three older Internet users (one heavy user and two moderate users). The age of heavy users ranged from 26 to 67, while the age of moderate users ranged from 39 to 75. All interviewees interacted frequently with their close ties face-to-face or through the telephone/mobile phone. Internet users also interacted online with close ties; in fact, all users emphasized the cheap and easy communication opportunities offered by the Internet. The older Internet users mostly used the Internet to communicate with relatives living close and far, whereas other participants mentioned relatives and close friends equally. For instance, Maria (age 67, retired) used Messenger to communicate with her son and grandson living abroad, Paulo (age 75, retired) used Skype to see his daughter in Portugal but also his son living in Spain and his other daughter in England, and João Nuno (age 67, retired) reports using Skype, email, and Instant Messaging to communicate with family living in the same city and with his son living in a different region. João Nuno was a former IT technician, so displayed a deeper knowledge of digital technology comparing to the other two older users.

**Table 8:** Types of Internet use & sociodemographics (interviewees).

	Pseudonym	Gender	Age	Education	Occupation
**Heavy**	Guinaldo	M	63	Undergraduate degree	Lawyer
	Clara	F	60	Graduate degree (Ph.D.)	Pediatrician
	Susete	F	54	Secondary education	Housewife
	João Nuno	M	67	Secondary education	Retired (former IT technician)
	Francisca	F	31	Graduate degree (Ph.D.)	Lecturer
	Cassandra	F	26	Undergraduate degree	Artist
	Daniel	M	31	Undergraduate degree	Journalist
**Moderate**	Paulo	M	75	Secondary education	Retired (former bank clerk)
	Pedro Lopes	M	45	Undergraduate degree	Flight attendant
	Maria	F	67	Undergraduate degree	Retired (former jurist)
	Marina	F	39	Secondary education	Assistant in a day care center
**Non-users**	Irene	F	85	Secondary education	Retired (former public servant)
	Sara	F	74	Primary education	Retired (former housewife)
	Fernando Jorge	M	83	Primary education	Retired (former construction worker)

Of the users, all except two reported a positive impact of the Internet on their interaction with strong ties; all, except three, reported a positive impact of the Internet on their interaction with weak ties. Those who did not report a positive impact, namely Daniel (age 31, journalist), Pedro Lopes (age 45, flight attendant) and Paulo (age 75, retired), indicated that the Internet did not change their interaction with social ties. Generally, most highlighted the positive effect of Internet use on their social lives: from facilitating a more frequent social interaction with close and weak ties to re-uniting with old friends online. For example, Maria (age 67, retired), explains the impact of Internet use on weak ties:

I mean it has in this aspect, people that I haven’t seen in a while, and that I’m not in permanent contact with, I mean weekly, I get once in a while an email asking how am I doing and I also send [an email back]. We send an email that we think it’s funny, so there’s always a hello, things like it was a letter. Before, we would write postcards, we would travel and send a postcard and say hi, and now the Internet serves for that.

Only two of the Internet users indicated having met a new person online. We found in the interviews a sense of inappropriateness or embarrassment of meeting people online or of admitting to it. The respondents answered negatively with non-linguistic signs of disapproval or would add expressions such as “I’m being honest. I haven’t met anyone online”. Thus, most of the interviewees’ online ties were offline ties. All reported the convenience of the medium to access various information and services. Some interviewees mentioned that the Internet allowed for multi-interaction, multi-tasking, spontaneity in social interaction (for example, a friend might show up online and an interaction might follow with no scheduled intention), and helped reduce social isolation and loneliness. For Maria (age 67, retired), the Internet:

(…) is very positive because of the openness that it brings to people, because people are not isolated, they don’t get lonely, because they always have that [the Internet] possibility…I don’t use it for that, but there are people that even meet online, I have one friend and she made two big friendships through the Internet and then they met, a man and a lady, and now they left the Internet and interact face-to-face. I think it has a lot of advantages in every aspect, in the daily life aspect, in the professional aspect, in the study aspect, of new knowledge or needs (…)

Only our older adults mentioned social isolation and loneliness throughout their interviews.

But interviewees also indicated negative aspects of Internet usage that may affect their social capital: while all showed concern with email overload and privacy issues, only interviewees in the 45–65+ age brackets reported addiction fears, such as spending too much time online. João Nuno (age 67, retired) encapsulates these main concerns:

There is a systematic search to interfere with our life, with suggestions…I don’t know, even provocations. If a person is not psychologically strong enough, even morally strong, it’s very complicated (…) And wasting time, sometimes we are there looking for nothing when we could have been doing something else…

In particular, non-users were widely vocal about the “dangers” of the Internet, indicating physical harm (such as visual impairments), pedophilia, and psychological and social consequences of offline displacement (i.e., the Internet replacing offline interaction and people being replaced by machines). These fears were fuelled by the media and did not relate to their personal experience. The positive aspects of the Internet for these non-users were its usefulness to access information and services. And as Sara (74, former housewife) put it: “the Internet shows you beautiful things”. Sara is a curious case, because she considered herself a non-user but used the Internet with the assistance of relatives (see discussion on ‘faux-users’, [70]) to contact her granddaughter living in Milan and to know more about the city:

My nephew showed me [online] Milan, where my granddaughter is (…) And I saw the Milan cathedral…and my granddaughter had already told me that it was beautiful. And so I said to my husband, if we had a computer at least we could see other beautiful things. We never go anywhere and so we could see beautiful things. And I thought it was really funny to hear the boy say to me ‘Grandmother, do you want to see Milan?’ And really from the words I could see there, he was really showing me Milan (…) we wouldn’t be so isolated of the world.

Sara was the only non-user that wanted to learn how to use the Internet, perhaps due to her indirect experience with the medium.

Finally, the interviews provided examples of mobilization of social capital, predominantly of expressive resources. Of the 14 interviewees, half indicated requesting assistance from ties in the past year: emotional (due to relatives passing away, work conflicts, and personal problems), practical (help accessing online information and publications), and financial aid (down payment for a house). The practical aid was mostly mobilized online for Internet users.

We also explored reciprocity; and all except one described helping ties, mainly relatives and close friends, through emotional support, practical assistance, specialized help (medical and legal advice), and financial aid. Despite some examples of instrumental resources (one of the interviewees was helping a friend to find a job; another was helping a friend and colleague with legal advice), most examples were of expressive resources (emotional support).

## Discussion

### Social affordances of the Internet

This study found that social capital is positively related to Internet usage but negatively related to age. Yet, different relationship patterns between these variables are captured by a significant interaction term. The interaction term between age and Internet usage has an effect on the likelihood of having low or high social capital. Without the interaction term, Internet usage–and not age–predicts social capital. The Internet might be, therefore, acting as a moderator: as Internet usage increases, the likelihood of having a high level of social capital increases. Concurrently, the Internet seems to counterbalance the negative relationship between age and social capital: older heavy Internet users had more probability of having high social capital than older non-users or light-users. Because we are working with cross-sectional data, a bi-directional relationship might also be at play: older adults with high levels of social capital might be more likely to use the Internet, but the strong interaction effect and its direction add to the complexity of this discussion. When we analyzed each dimension of social capital we found similar patterns, although not the interaction term: Internet usage is positively related to bonding and bridging, whereas age is negatively related to bonding, bridging, and resources.

Online services, such as email, SNSs, and IM, did not predict social capital, invalidating our assumption that more social-driven online use could affect social capital. As Erickson [42] concluded, Facebook was not used for substantial interaction among older adults although provided awareness of what was going on with relatives and friends. It may be that Internet usage is more than the sum of its parts, or that we were not able to grasp the intricacy of these social media with a dichotomous variable. The Internet, in general, seemed to allow daily users to maintain and reinforce their social relationships (e.g., three-quarters of the survey respondents said it made it easier to be in touch with relatives and friends), as well as to mobilize resources. The social affordances of the medium–i.e., the Internet’s convenience, social connectivity, ubiquity, and social cues that create varied opportunities for interaction–seem to be fruitful for production, accrual, and mobilization of social capital [71–73]. Within this social affordances perspective, connecting technology, agency, and social structures, the Internet offers: 1) a range of synchronous and asynchronous communication opportunities with different ties, 2) the possibility of meeting new ties, 3) the potential to re-unite old ties (such as school friends), and 4) the ability to manage the length and the scope of social interactions, while doing other things at the same time.

The interviews strengthen our understanding of these affordances by showing that the Internet facilitated regular contact with close and weak ties across age groups: the Internet was embedded in most of our interviewees’ daily lives, being frequently used for short and long social interactions. It also allowed interviewees to contact ties living close or far and to re-connect with old friends. In particular, for our older adults, the Internet was an important medium in the context of transnational families, bringing together children and grandchildren living abroad. Additionally, the Internet was used to mobilize social capital, through accessing information, psychological support, or practical help provided by a tie, or to coordinate social agendas.

Taken together, these results support and extend the scarce research on Internet use and social capital in old age and across adulthood [40,41]. But while the literature suggests that meeting people online could reinforce social capital by forming ties, less than half of our Internet users reported meeting people online. Age was also negatively correlated with meeting new people online. Likewise, only two of the interviewees reported meeting people online and the remaining interviewees displayed a sense of impropriety when discussing it. This may relate to the age of our respondents (+18) or to Portuguese idiosyncrasies. Furthermore, less than a third of the survey respondents had online ties (people that they never met in person)–online ties seemed to transition rapidly to offline ties, as shown in previous research: when people meet online and establish a relationship, they often meet offline [18].

### Matthew effects: Cumulative social advantage/disadvantage

While the social affordances of the Internet help explain its positive relationship to social capital, lifecourse changes in social networks may explain the negative relationship between social capital and age. Research shows that the number of strong ties and the extent of friendship declines with age due to widowhood, retirement, physical and functional decline, loss of mobility, and other lifecourse circumstances [74–76]. Our findings showed a negative correlation between age and the number of close friends people have, along with a greater reliance of older adults on relatives rather than friends for social connectedness and resources. Different life stages might affect the availability of psycho-social-economic resources (e.g., older people would not require help to find a job as other age groups) and the ties available to access these resources. Although older people tend to have a stable network of relatives, their network is often reduced with the death of a member, health decline, moving to care homes, etc. [75]. Moreover, older age is associated with older networks both for men and women [77]. So more than personal choice or relational skills, there are strong structural constraints for close relationships and sociability in old age [74,76,78]. For instance, social network types in old age, such as the family-focused, friend-focused, diverse (mixed ties), congregant (faith-based), and restricted (limited extent of social ties), can affect social capital differently [79,80]. Older adults embedded in networks with a wider range of social ties (e.g., diverse, friend, and congregant) fare better in terms of well-being, reporting greater happiness and less loneliness and anxiety [80]. Restricted access to broader social networks, loss of ties, and infrequent social interaction means that older people have fewer opportunities to accrue resources. Our results indicate that younger people are frequently at an advantage: heavy young users are more likely to have a higher level of social capital than older heavy users. Our data shows that a 20-year-old person is 29 per cent less likely to have a high level of social capital if a non-user, whereas a 70-year-old person is 70 per cent less likely to have a high level of social capital if a non-user.

However, our data also demonstrates that those who used the Internet daily are more likely to have high social capital. Older heavy Internet users were better off than moderate, light, and non-Internet users, regardless of age. It seems the Internet has a *buffering* element that counterbalances the negative association between age and social capital. But older adults are still less likely to adopt the Internet when comparing to other age groups, and education is a top determinant of Internet use among older adults [81]. Thus, only those highly educated would benefit from that *buffering* factor. While the Internet seems to be helping maintain, accrue, and even mobilize social capital through its social affordances, it also reinforces forms of social inequality, particularly in terms of age and education. Therefore, the *Matthew effect* or the cumulative advantage theory offers one framework for understanding the role of the Internet and its relationship to social capital. The “Matthew effect” was coined by sociologist Robert K. Merton (p58 in [82]) to illustrate processes of misallocation of credit and rewards to scientists. Merton takes the expression from the St. Mathew’s Gospel (‘Parable of the Talents’) to theorize cumulative social advantage:

(…) the Matthew effect consists in the accruing of greater increments of recognition for particular scientific contributions to scientists of considerable repute, and the withholding of such recognition from scientists who have not yet made their mark (p58 in [82]).

This theoretical perspective is, nonetheless, a principle operating “in many systems of social stratification to produce the same result: the rich get richer at a rate that makes the poor become relatively poorer” (p62 in [82]). Despite criticisms on the choice of the term, it was quickly popularized to describe accumulated gains having a wide scientific application as a cumulative advantage/disadvantage theory [83,84]. Although early research on Matthew effects focused on inequalities in science, researchers have been uncovering Matthew effects on lifecourse processes and in several domains, from education to economic systems [84–86]. In fact, Merton revisited the concept in 1988 showing its use in different disciplines and reinforcing that:

The concept of cumulative advantage directs our attention to the ways in which initial comparative advantage of trained capacity, structural location, and available resources make for successive increments of advantage such that the gaps between the haves and the have-nots in science (as in other domains of social life) widen until dampened by countervailing processes (p606 in [87]).

Because the Matthew effect lens captures broad inequality-generating processes, it is a useful overarching framework to help describe social capital dynamics and the revealed relationship between social capital and Internet usage: advantage begets further advantage, and disadvantage begets further disadvantage. Heavy users had more probability than any other type of users of having high social capital, and heavy users were more likely to be younger and highly educated. Moreover, our findings show that older people had less probability of having high social capital, independently of the frequency of Internet usage, even though older heavy users were better off than older non-users. Recent research on Internet use and socioeconomic status adds to this approach: growing up in advantaged families and having higher income in older adulthood increases the odds of using the Internet daily [88].

Matthew effects also relate to what Merton [89] defined as manifest and latent functions (intended/unintended) of social phenomena: most digital technologies are developed by educated adults to offer what developers perceive to be convenient and useful options for end-users (manifest functions). However, these end-users are often young adults and adults–users with some level of digital knowledge, technology acceptance, technology needs, and disposable income. Intrinsically, as research shows [4], older adults are usually excluded from the design process of mainstream digital technology (latent functions). Of course these functions can be positive as well: non-educated older adults might be benefiting from digital exclusion, avoiding potential social risks of online use. Nonetheless, research has been showing that the positive social and psychological outcomes of digital inclusion overcome negative ones [16]. Another Matthew effect that interplays with our results is the cumulative cycle of technology development [84,90]–although innovation is far from a linear model [91], technology is mostly built upon previous technology and evolves rapidly, meaning that not only does “technology [beget] more technology” (p258 in [92]) but digital skills need to be constantly updated. This adds another layer of complexity to functional lifecourse barriers of Internet use among older adults.

Despite its explanatory value in describing and interpreting disparities in the distribution and accumulation of resources across different areas (in relative or absolute terms), the Matthew effect was developed as a theoretical and analytical framework and not “an iron law of nature” (p36 in [84]). Merton’s work to conceptualize the Matthew effect was based on qualitative research; the “effect” was intended as a principle or factor [82,87]. As such, although quantitative approaches to Matthew effects have been the norm, qualitative studies continue to be critical in disentangling cumulative advantage/disadvantage processes in everyday life [83]. The “countervailing” factors that can mute Matthew effects (p617 in [87]) are ceiling or floor effects, intergenerational dispersion of resources, social policy, etc. [84,87,93]. For example, the ceiling effect is often considered when discussing older adults and the Internet, since it is expected that as generations change, the increase of users in industrialized societies will slow down reaching an asymptotic limit (100%). However, this argument does not take into account the fast development of the Internet, the social shaping of technology (i.e., social and ideological choices embedded in the design and implementation of any technology), the changing demands on digital literacy and motor and cognitive abilities, diverse socioeconomic dimensions, and differences across countries.

In our case, the only finding inconsistent with this Matthew effect framework is the difference between non-Internet users and light users: non-Internet users had better chances of having a high level of social capital than light users. Still, we can hypothesize that these light users might face other structural disadvantages including, for example, personality traits (our light users may be more introverted, or less open, sociable, and agreeable than non-users), specific social circumstances (such as an Internet newbie that is still learning how to use the medium and experiences a mild social anomie), and types of Internet usage (more oriented for individualistic activities than social ones). Light users are also underrepresented in our sample, which can be skewing the results.

Regarding other predictors, compared to widows, single people had more odds of a high level of social capital. This probably relates to the fact that widows lost close social ties and are more likely to be older people, meaning generally a low social capital. Single people were more likely to be younger and have more ties and social capital. People that lived alone had lower odds of having high social capital, comparing to households with a higher number and/or diversity of ties. Perhaps bigger or more diverse households signify more availability of resources and, hence, of social capital.

In closing, as different forms of capital interconnect, advantages in social capital and in Internet use not only lead to advantages in social resources and digital inclusion, but also bring further cultural, political, and psycho-socio-economic advantages.

## Conclusion

This article contributes to an understanding of social capital and Internet use across age groups and particularly in old age. This is an important topic for aging and technology studies due to the role of the Internet on social participation and connectedness, and to the significance of social capital on psychosocial well-being and resource mobilization in later life. Although research on their relationship has been scant, our mixed-methods study represents a step in addressing this gap, adding to the wealth of literature on young adults, and to the scarce research that looked at Internet and social capital among older adults.

Our findings show a strong interaction effect between Internet and age on social capital, providing support for the theory of cumulative advantage or Matthew effects: younger Internet users were better off than older Internet users, but older daily Internet users fared better than non-users and other types of users. The Internet, then, seems to be a moderating factor buffering for the negative relationship between age and social capital. Education, though, predicts Internet use by older adults [81], which means that only those highly educated can profit from that buffering element. The Internet seems to, simultaneously, have a positive effect on social capital and reinforce accumulated social advantage. The Matthew effects within this relationship help to describe a pattern of growing inequality that links digital and social exclusion, including the relationship between social capital, the Internet, and age, but also the cycle of technology development and use. Despite the strong interaction effect, we cannot be certain of the direction of this relationship: it may be that older adults with high social capital are more likely to use the Internet. We suspect that this is a reciprocal feedback process: those with strong levels of social capital often use the Internet to keep in touch and access resources; those who use the Internet daily enhance their existing relationships and resources. Our interview data added to this relational understanding.

Findings also supported the social affordances of the Internet in assisting with maintenance of ties and resource mobilization. These affordances can be crucial for older adults–especially for those with health-related mobility issues, living alone or transitioning to institutions–as a way of reinforcing their assets for instrumental and expressive actions and enhancing their social and psychological connectedness. As reported, our older interviewees were distinctly concerned with social isolation and loneliness. The Internet can offer opportunities for social interaction in old adulthood to help lessen social isolation and loneliness (and its harmful effects, namely depression, cognitive and functional decline, morbidity, death, and civic disengagement among older adults) [2,94–96]. The Internet can also provide access to relevant information and services for older adults, which can improve their independence, well-being, and social participation. In a society progressively mediated by the Internet, digital exclusion (not having access or the literacy to use digital technologies) is emerging as a prominent form of social exclusion and inequality [97,98]. For instance, the gradual migration of public services to online-only socially excludes those who are digitally-excluded. As our results suggest, the Internet and social inequality are not isolated entities; they shape each other dynamically. As such, we need to identify functional and attitudinal barriers to digital access and literacy and develop initiatives to tackle digital exclusion that go beyond equipment provision and include modules on critical digital literacy. Critical digital literacy is vital to facilitate a knowledgeable, safe, effective, and efficient use of the Internet, reducing potential negative outcomes of Internet use among older adults such as fraud, scams, privacy issues, etc. [97,99].

Although we move forward our understanding of Internet use, social capital, and age, it is not without limitations. First, these findings are limited by a cross-sectional design. Second, while Portugal shares some demographic and social capital patterns with other European countries [48], our results are limited by the possible socio-cultural idiosyncrasies of the Portuguese and the Lisbon context. Third, we did not have indicators of perceived health status, which could affect Internet use. Previous studies have found that health problems can facilitate or inhibit Internet use: for instance, some mobility-related impairments enhance Internet adoption and usage, whereas dementia curbs it [5]. Our selection criteria excluded people with dementia or any cognitive impairment that could render them unable to consent, but relied on self-assessment (often influenced by self-presentation efforts and stigma management). We also recorded visible impairments that could inhibit Internet use–but this was not explored in-depth with our participants. Fourth, we are limited by the instruments and indicators selected; for example, the online vs. offline social capital sub-scales were inapplicable. Finally, although our analysis of specific social media (email, social networking sites, and instant messaging services) did not yield any significant results, these were measured as dichotomous (use/non-use) variables. Different types of use of these social media might generate other results.

Despite these limitations, our approach and results have implications for future research on social and digital stratification in a lifecourse perspective. They are also relevant for policy-informed initiatives to enhance social connectedness, participation, and social capital, as well as to bridge the digital divide among older adults [11,12,33,81]. Lastly, we hope that these findings can spur interest in dynamics of age for technology studies and in the role of digital technologies in social stratification in later life. Future research should examine types of Internet usage and social capital in a longitudinal perspective. Exploring health status, psychosocial abilities, and personality traits would expand our understanding of the relationship and outcomes of Internet use/non-use and social capital in old age.

## Supporting information

S1 File Survey dataset. (CSV)

S2 File Letter from CAPP’s ethics committee, university of Lisbon. (PDF)
