# Supplementary material for: Social capital and Internet use in an age-comparative perspective with a focus on later life
Source: PLoS One. 2018 Feb 26;13(2):e0192119. doi: 10.1371/journal.pone.0192119 (PMC5826529; doi:10.1371/journal.pone.0192119)
Supplement: S2 File — (PDF) [file pone.0192119.s002.pdf]

CAPP
Centro de Administração
e Políticas Públicas

To whom it may concern, The study 'Social Capital and Internet Use', which this manuscript is based upon, did not require formal ethics approval by the University of Lisbon, the research center affiliated with the authors (CAPP), or the funding agency (FCT, Portuguese national council for science, research, and technology). This was a cross-sectional, observational, social science project based on low sensitive issues, therefore not presenting any significant risk level to involve institutional approval according to the University and Portuguese regulations. However, the research proposal, which comprised an ethics section, was fully approved by the University doctoral committee. In addition, although not requested, procedural ethical requirements were strictly followed including informed consent and anonymity and confidentiality procedures as stated by the authors in their manuscript. While the Declaration of Helsinki (DoH) is not legally binding and was developed for the medical community, the procedures followed by the authors align with the ones defined in the DoH.

The President of CAPP – Centre of Public Administration and Public Policies,

Paulo Castro Seixas
